# Supplementary material for: Health service research definition builder: An R Shiny application for exploring diagnosis codes associated with services reported in routinely collected health data
Source: PLoS One. 2023 Jan 12;18(1):e0266154. doi: 10.1371/journal.pone.0266154 (PMC9836275; doi:10.1371/journal.pone.0266154)
Supplement: Attachment — Submitted filename: PLOS ONE Response to Reviewers.docx [file pone.0266154.s001.docx]

## Journal requirements

> Please ensure that your manuscript meets PLOS ONE’s style requirements, including those for file naming.

We have made the following formatting changes:

- Style of heading 1
- Labeling of figures
- Table titles
- Updated reference style (Vancouver, square brackets)
- The funding and conflict of interest statements have been removed from the acknowledgements section.

> Could you please provide further details on why your study is exempt from the need for approval and confirmation from your institutional review board or research ethics committee (e.g., in the form of a letter or email correspondence) that ethics review was not necessary for this study? Please include a copy of the correspondence as an "Other" file.

We have uploaded this correspondence regarding the ethics review process from the WCG IRB (formerly the New England IRB). The aims for this study was part of a larger project at the Lown Institute (previously exempt from IRB review), and so we submitted an amendment with these study aims to the WCG IRB who deemed that the additional aims did not change the exemption status.

The exemption was applied since we use de-identified claims data, which we have requested and received approved and secure access from the Centers for Medicare & Medicaid Services’ Research Data Assistance Center.

> Please provide additional details regarding participant consent. In the ethics statement in the Methods and online submission information, please ensure that you have specified what type you obtained (for instance, written or verbal, and if verbal, how it was documented and witnessed). If your study included minors, state whether you obtained consent from parents or guardians. If the need for consent was waived by the ethics committee, please include this information.

We have added a statement in the Methods section (page 4 lines 1-2).

> Please note that PLOS ONE has specific guidelines on code sharing for submissions in which author-generated code underpins the findings in the manuscript. In these cases, all author-generated code must be made available without restrictions upon publication of the work.

We have supplied the code for both the SAS and R application on GitHub (link provided in the text).

Regarding the minimal data sets and author generated codes for the tables:

- Table 2 was compiled from results that were output from running the SAS macros supplied in the above Github repository (specifically hsrdef_run.SAS).
- Table 3 was based on the saved output data set from carrier claims with CPT code 29877. This data set has been added to a folder in the Github repository titled (‘minimum_dataset_paper’) and labeled ‘results_table3_carrier29877.csv’.
- Table 4 results were compiled by the SAS code we have now added to the minimum_dataset_paper (called hsrdef_run_urinalysis_check.R). The datasets containing the important diagnosis codes listed in the table have also been added to this table (results_table4_carrier81001.csv, results_table4_carrier81003.csv, results_table4_outpatient81001.csv, results_table4_outpatient81003.csv).
- Table 5 was created using the data set output from the SAS application with inpatient claims for procedure codes 0SG0x. This data set has been added to the minimum_dataset_paper folder (results_table5_inpatient0SG0.csv).

The above description of the minimum data sets related to the paper have been added to the Github repository README file.

> Please remove any funding-related text from the manuscript and let us know how you would like to update your Funding Statement. Currently, your Funding Statement reads as follows:

> “The authors received funding from Arnold Ventures LLC for this project (ID 2004079) (<https://www.arnoldventures.org/>). The funders had no role in study design, data collection and analysis, decision to publish, or preparation of the manuscript.”

We have removed the funding statement from the text. The funding statement above is correct.

RE Competing Interest. The online competing interest statement should read:

I have read the journal's policy and the authors of this manuscript have the following competing interests: AE reports receiving personal fees from the Australian state government health departments – Victoria, Queensland, South Australia, as well as the Australian Department of Veterans Affairs, Medibank Ltd, Private Healthcare Australia, and the Australian Defence Force Joint Health Command and grants from the National Health and Medical Research Council outside the submitted work. AE is also on the board of the New South Wales Bureau of Health Information. This does not alter our adherence to PLOS ONE policies on sharing data and materials.

> Please include your full ethics statement in the ‘Methods’ section of your manuscript file. In your statement, please include the full name of the IRB or ethics committee who approved or waived your study, as well as whether or not you obtained informed written or verbal consent. If consent was waived for your study, please include this information in your statement as well.

This has been added to the Methods section (see above).

## Reviewer 1

Thank you to Reviewer 1 for their time and comments on our paper.

> Overall this problem has been addressed repeatedly in the literature. This paper uses a SAS procedure, HPSPLIT, at its core to address the problem. This is indeed a useful function in SAS, but obviously has been invented already and the paper describes a handful of workflow approaches to using the procedure and uses R (rather than the SAS built in visualizations) for showing the results.

In this paper, we describe our methods and developed tools for building thorough health service indicators. This is useful and important for other researchers developing service measures (whether related to low-value care or otherwise). We have not come across similar approaches or tools described in the literature. The need for such a solution has become clearer to us after our own internal audits on published indicators showed frequent or important diagnosis codes in claims data had sometimes not been included. The benefit of using R is due to the shiny package, which is not available in SAS, and creating a dashboard that can be used by non-coders and those who do not have direct access to claims data.

> The problem to be addressed is put in a very restrictive context and those who are not familiar with using claims data would have trouble seeing the value, would recommend in future papers that the perspective be broadened.

We agree with Reviewer 1 that this - low-value service indicators - is a somewhat niche problem. A large number of health service research studies, however, do use administrative data and create cohorts based on service and diagnosis codes (as mentioned in our introduction). We have written our paper and built our tool for a research audience, where we assume at least one collaborator is familiar with claims data and the processes involved in creating these initial indicators/cohorts.

> There are many arbitrary decisions to accommodate technical specifics, such as using only the first 4 digits of ICD codes which are not semantically justified.

This is a challenge that we came across while developing this process, so thank you to Reviewer 1 for raising this issue. Ultimately this is a process and tool for data exploration and visualization. Like many visualization approaches, decisions around summarizing data have to be made. A simple example is choosing the number of bins to use to display data in a histogram.

We decided on using a limit of four characters for ICD diagnosis codes because of the organization of diagnosis information in the ICD codes. For example, M17.1 describes “Unilateral primary osteoarthritis”, M17.11 describes “Unilateral primary osteoarthritis, right knee” and M17.12 describes “Unilateral primary osteoarthritis, left knee”. The actual informative condition for the purpose of this exercise is unilateral primary osteoarthritis, which just requires four characters of the ICD codes.

> The reporting of only outcome statistics for the specific samples used for training is a serious limitation

Thank you to Reviewer 3 for pointing this out. We had originally decided to report only on the original training set since our goal was to select the diagnosis codes ‘important’ to this sample. After revisiting the paper, we decided to report the sensitivity, specificity and misclassification rates on a validation sample of claims in Table 2 (and have made updates to the results on page 9 to reference these results).

## Reviewer 2

Thank you to Reviewer 2 for their time and comments on our manuscript.

> I think that the tool is not easy to use. One issue is the development of a SAS to R workflow. I suggest to include a default SAS output to upload when a user opens the app.

We have revisited the R tool and package and have taken the opportunity to make a number of improvements, including Reviewer 2’s useful suggestion to have an option to upload an example data set. Our other improvements include:

- Including checkboxes for Include/Exclude options for codes
- Adding a row ID column so sorting is easier
- Updating the label input as a type box/drop down menu so previous labels can be saved
- Adding colors to the action buttons

> The documentation should be richer with more details.

As well as the changes made above, we have included more instructions on the application when the user opens it. All of these updates can be observed in the Github repository.

## Reviewer 3

> I thank the authors for investigating such an important subject.

We thank Reviewer 3 for their time and thoughts on our manuscript.

> The structure of the manuscript was not organized properly, and it was hard to follow. The Materials and Methods section should be before the Results section.

We had originally organized in this way to follow some previous PLOS ONE publications that we had found useful in understanding the purpose/outputs (the results) prior to the details of the Materials and Methods. We do take Reviewer 3’s point, however, and have updated the text so the Materials and Methods are before the Results.

> The data described that patients younger than 65 were excluded from the study, so how can the results be generalized to people of all ages?

Our goal was to build indicators for services provided to people 65 years and older, and never to generalize to data on people of all ages. Users working with other populations will have to change this age restriction. We have now noted this in the discussion section (p18, lines 1-3):

“We also restricted our claims data to services provided to patients aged 65 and over. If users have a different population in their study, they will have to edit this step in the SAS code, in order to find diagnosis codes relevant to their population.”
